# Supplementary material for: Dramatic impact of future climate change on the genetic diversity and distribution of ecologically relevant Western Mediterranean Carex (Cyperaceae)
Source: PeerJ. 2022 May 31;10:e13464. doi: 10.7717/peerj.13464 (PMC9165605; doi:10.7717/peerj.13464)

Supplementary Files

Supplemental Fig. S1. Distribution of pairwise differences among AFLP phenotypes in *C. reuteriana* and *C. panormitana* inferred with $AFLP_{DAT}$.

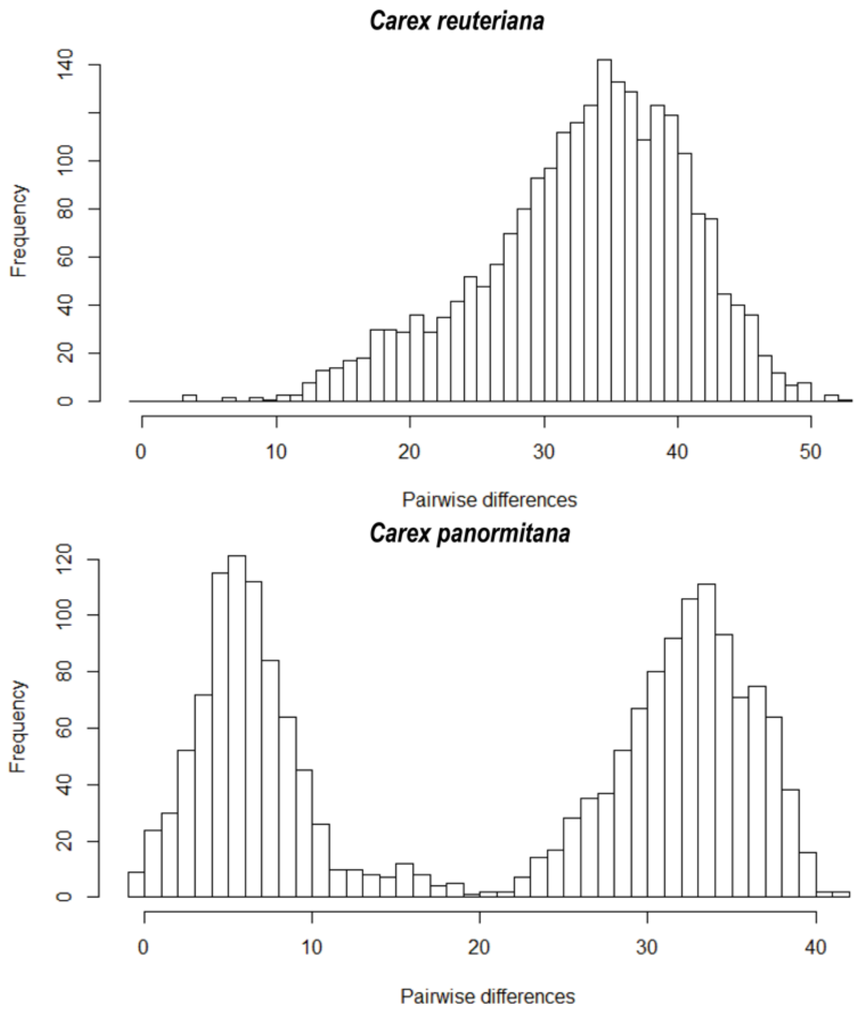

Supplemental Fig. S2. Mantel test, correlation coefficient and scatterplot showing the relationship between pairwise genetic distance among *C. reuteriana* subsp. *reuteriana* populations and the geographical distances among them.

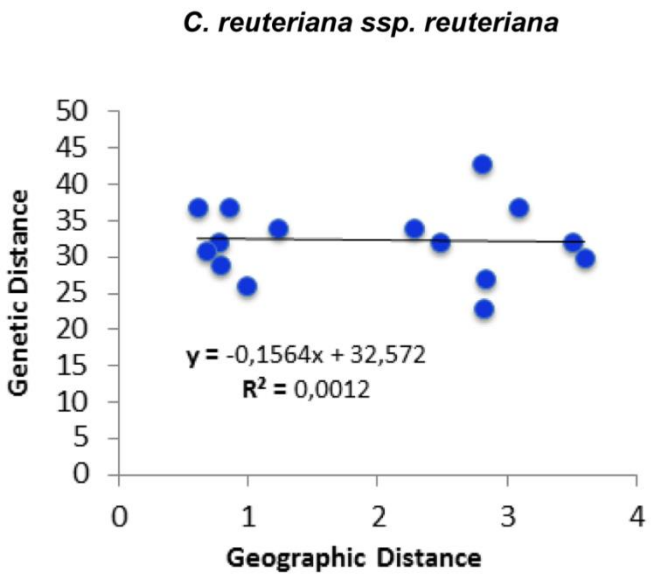

Supplemental Fig. S3. Predicted changes of potential distribution inferred by Biomod comparing between present and 2041-2060 period under RCP2.6 and RCP8.5 climatic change scenarios for *C. reuteriana* subsp. *reuteriana*, *C. reuteriana* subsp. *mauritanica* and *C. panormitana*. Future projections represent the consensus SDM averaged across six GCMs. Percentages indicate the proportion of potential range inferred to be gained, lost, or to remain stable in the future in comparison with the present, according to the following colour scheme: Red areas are currently suitable areas predicted to be lost, orange areas are currently suitable areas projected to remain stable, and green areas are currently unsuitable areas projected to become suitable.

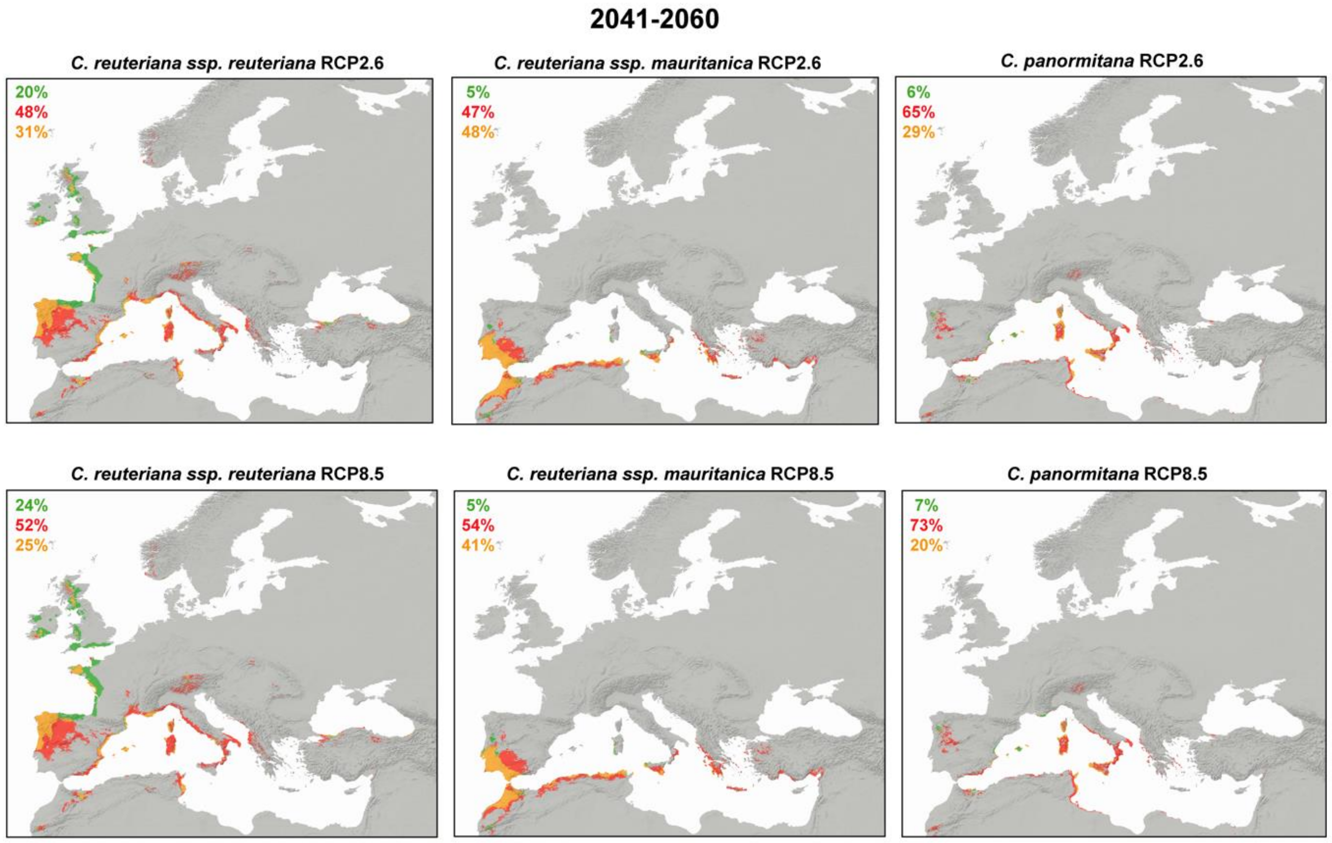

Supplemental Fig. S4. Representation of the environmental space of each taxon (*C. reuteriana* subsp. *reuteriana*, *C. reuteriana* subsp. *mauritanica* and *C. panormitana*) at present and 2081-2100 under the optimist climate change scenario (RCP2.6). Plots display the values for four bioclimatic variables as retrieved by Principal Component Analysis (PCA) and their contributions in both axes. The arrows indicate the variable's contribution to the PCAs.

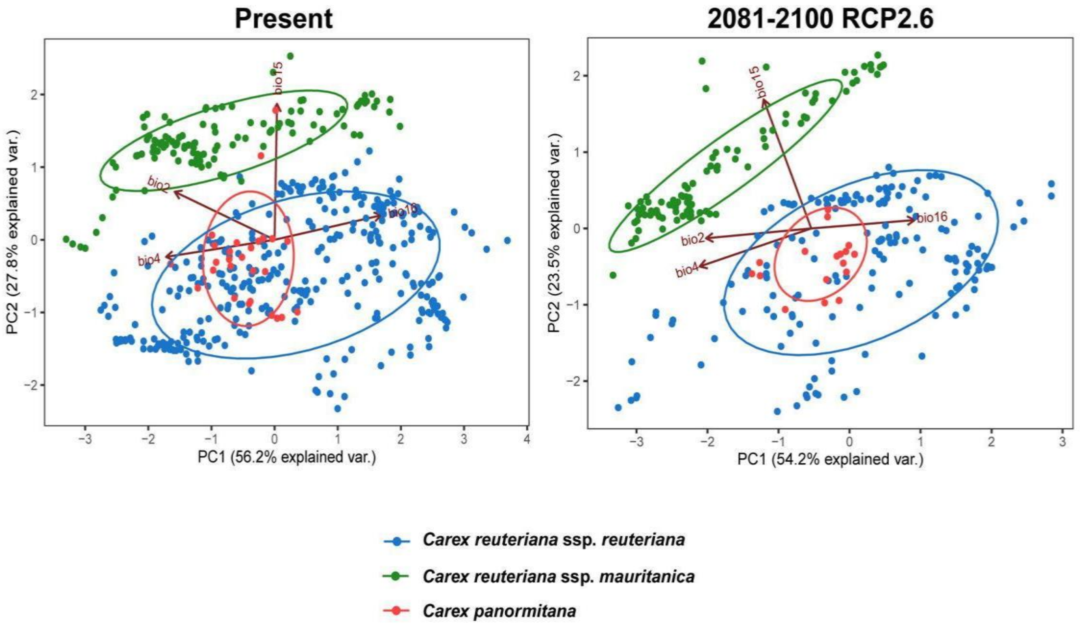

Supplement: Supplemental Information 2 — Fig. S1. Distribution of pairwise differences among AFLP phenotypes in C. reuteriana and C. panormitana inferred with AFLPDAT. Fig. S2. Mantel test, correlation coefficient and scatterplot showing the relationship between pairwise genetic distance among C. reuteriana subsp. reuteriana populations and the geographical distances among them. Fig. S3. Predicted changes of potential distribution inferred by Biomod comparing between present and 2041–2060 period under RCP2.6 and RCP8.5 climatic change scenarios for C. reuteriana subsp. reuteriana, C. reuteriana subsp. mauritanica and C. panormitana. Future projections represent the consensus SDM averaged across six GCMs. Percentages indicate the proportion of potential range inferred to be gained, lost, or to remain stable in the future in comparison with the present, according to the following colour scheme: Red areas are currently suitable areas predicted to be lost, orange areas are currently suitable areas projected to remain stable, and green areas are currently unsuitable areas projected to become suitable. Fig. S4. Representation of the environmental space of each taxon (C. reuteriana subsp. reuteriana, C. reuteriana subsp. mauritanica and C. panormitana) at present and 2081–2100 under the optimist climate change scenario (RCP2.6). Plots display the values for four bioclimatic variables as retrieved by Principal Component Analysis (PCA) and their contributions in both axes. The arrows indicate the variable’s contribution to the PCAs. [file peerj-10-13464-s002.pdf]
